# Dr. J. Talyor

**Published:** 1856-06

**Authors:** A. Blake

**Affiliations:** St. Louis, Mo.


### Correspondence.

Dr. J. Talyor,—Dear Sir:—The following case recently came under my observation. If you think it worth a place on some page of your valuable Journal, it is at your disposal.

We read of teeth having been extracted, and then replaced; also of persons having a defective incisor extracted, and a like tooth without the defect taken from the jaw of another person, and transplanted in their own, but this practice like many other things in this age of progress is among the things that were. It was only called to mind by the case I have to relate.

Mrs. S., about forty years of age, called at my rooms a short time since to consult me in regard to a tooth. She had worn a plate about seven years with the four superior incisors. They were attached on the right to the first bicuspid, on the left to the first molar. In February last, the bicuspid to which the plate was attached came out. It had been slightly loose for some time, occasioned by absorption. She however, re-placed the natural tooth, also the artificial piece, and in this way has worn the whole for the last four months. There is no union between the fang of the natural tooth and its socket, as on removing the plate it sometimes comes out; but is easily and without pain replaced.

Respectfully yours,

A. BLAKE.

St. Louis, Mo. June 6th, 1856.

We very often see plates worn by a single clasp, one having given way, and indeed, clasp teeth are sometimes worn as suction plates, after both teeth to which the clasps had been attached gave way. Use will enable a person to wear teeth that at first would not or could not be kept in the mouth. We knew a lady who wore five or six upper front teeth for years, and held them in the place by the lip and tongue.

